# Genomic Predictors of Platinum Resistance and Survival in High-Grade Serous Ovarian Carcinoma: Insights from an Explorative Targeted Next-Generation Sequencing Analysis

**DOI:** 10.3390/cancers18091390

**Published:** 2026-04-28

**Authors:** Carmela De Marco, Valentina Rocca, Simona Migliozzi, Claudia Veneziano, Francesca Gualtieri, Annamaria Cerantonio, Tahreem Arshad Butt, Gianluca Santamaria, Maria Teresa De Angelis, Annalisa Di Cello, Roberta Venturella, Fulvio Zullo, Giuseppe Viglietto

**Affiliations:** 1 Molecular Oncology Laboratory, Department of Experimental and Clinical Medicine, “Magna Graecia” University, 88100 Catanzaro, Italy; valentina.rocca@unicz.it (V.R.); veneziano@unicz.it (C.V.); francesca.gualtieri002@studenti.unicz.it (F.G.); cera90anna@hotmail.it (A.C.); tahreemarshad.butt@studenti.unicz.it (T.A.B.); gsantamaria@unicz.it (G.S.); mariateresa.deangelis@unicz.it (M.T.D.A.); viglietto@unicz.it (G.V.); 2 Interdepartmental Center of Services (CIS), Omics and Biobank, “Magna Græcia” University of Catanzaro, 88100 Catanzaro, Italy; 3 Medical Genetics Unit, Renato Dulbecco University Hospital, 88100 Catanzaro, Italy; 4 Clinical Genomics and Therapeutics Division, Translational Genomics Research Institute (TGen), Phoenix, AZ 85004, USA; smigliozzi@tgen.org; 5 Unit of Obstetrics and Gynecology, Department of Experimental and Clinical Medicine, “Magna Graecia” University, 88100 Catanzaro, Italy; annalisa.dicello@gmail.com (A.D.C.); venturella@unicz.it (R.V.); zullo@unicz.it (F.Z.)

**Keywords:** high-grade serous ovarian carcinoma, platinum resistance, next-generation sequencing, DNA repair, transcriptional regulation, epigenetic modification, oncogenic signaling, prognostic biomarkers

## Abstract

**Background:** High-grade serous ovarian carcinoma (HG-SOC) remains the most lethal gynecological malignancy, largely due to intrinsic or acquired resistance to platinum-based chemotherapy. Although large-scale sequencing studies have delineated the genomic landscape of HG-SOC, clinically actionable biomarkers predictive of platinum response and outcome are still lacking. This study aimed to identify genomic alterations associated with platinum sensitivity, resistance, or refractoriness, and to assess their prognostic relevance. **Methods:** Tumor DNA from 24 HG-SOC patients with optimal cytoreductive resection, classified as platinum-sensitive (*n* = 9), platinum-resistant (*n* = 8), or platinum-refractory (*n* = 7) underwent targeted next-generation sequencing of 409 cancer-associated genes. Somatic variants were filtered and classified for oncogenicity using established criteria incorporating predicted functional impact, REVEL scores, and population allele frequencies. Associations between mutational profiles, platinum response, and overall survival (OS) were evaluated using Kaplan–Meier and Cox regression analyses. Key findings were validated in the TCGA ovarian serous carcinoma (TCGA-OV) dataset using survival analyses. **Results:** A total of 1367 protein-altering somatic variants across 301 genes were identified. While *TP53* mutations were ubiquitous, platinum-resistant and platinum-refractory tumors showed enrichment of pathogenic alterations affecting DNA repair, transcriptional regulation, epigenetic modification, and oncogenic signaling, including *FANCA*, *ATF1*, *MAF*, *NCOA2*, *PIK3CA*, and *TET1*. Mutations in these genes were associated with reduced overall survival in exploratory analyses (median 2.5–9 months vs. 27.5–45 months). Multivariate analysis identified *FANCA* and *ATF1* as potential independent predictors in exploratory modeling. In the TCGA-OV cohort, patients harboring pathogenic variants in a multi-gene panel derived from this study (excluding *BRCA1/2*) exhibited significantly worse survival compared with both *BRCA1/2*-mutated cases and the overall cohort. **Conclusions:** This exploratory study identifies a set of genomic alterations converging on transcriptional and epigenetic regulation, DNA repair, and oncogenic signaling that are associated with platinum resistance and adverse prognosis in HG-SOC. Independent validation in TCGA supports the potential clinical relevance of this mutational signature. These findings warrant further validation in larger prospective cohorts and functional studies to clarify their role as biomarkers of aggressive disease and therapeutic vulnerability.

## Simple Summary

High-grade serous ovarian carcinoma (HG-SOC) is primarily treated with platinum-based chemotherapy; however, many patients experience early relapse or primary resistance, and robust predictive biomarkers remain lacking. As a result, clinical management is largely uniform despite pronounced biological heterogeneity. In this study, we identified gene alterations that are more frequent in platinum-resistant and platinum-refractory tumors and are associated with poorer survival. These alterations affected genes involved in DNA repair, epigenetic regulation, transcriptional control, and oncogenic signaling. Importantly, our findings were independently validated in the TCGA ovarian cancer cohort, where a multi-gene panel derived from our study identified a subgroup of patients with aggressive disease and unfavorable prognosis, distinct from *BRCA1/2*-mutated cases. Collectively, our results suggest that expanding genomic profiling beyond BRCA status may improve risk stratification and help identify patients who could benefit from alternative or more intensive treatment strategies.

## 1. Introduction

Epithelial ovarian cancer (EOC) is the most common type of gynecological malignancy [1,2], and is the tumor with the highest mortality rate of all gynecological malignancies [3]. EOC patients have an overall low five-year survival rate (approx. 30%) [4], as the diagnosis is often only made at an advanced stage—due to a lack of specific symptoms—and most tumors are (or become) resistant to platinum-based chemotherapy [5,6].

High-grade serous ovarian carcinoma (HG-SOC) accounts for ~70% of EOC cases and is characterized by frequent *TP53* mutations, genomic instability, and a poor prognosis. Despite optimal cytoreductive surgery and standard first-line chemotherapy with carboplatin plus paclitaxel, up to 80% of patients relapse, and more than half develop platinum-resistant disease within 6–12 months [7,8].

Based on the response to platinum drugs, patients are classified as: (i) sensitive if they relapse at least 12 months after treatment; (ii) resistant if they relapse within 6 months of starting therapy; (iii) refractory if they already had progressive disease at the start of therapy [9,10]. The prognosis for platinum-refractory patients is dismal, with median survival often below 12 months [11]. Current second-line therapies, such as pegylated liposomal doxorubicin or topotecan, provide only limited benefit [12]. Overcoming platinum resistance therefore remains an urgent clinical priority.

Several mechanisms contribute to platinum resistance, including: (i) increased drug efflux via ATP-binding cassette transporters (ABC transporters) [13,14]; (ii) decreased intracellular drug accumulation via modulation of copper transporters [15,16]; (iii) increased DNA repair capacity via homologous recombination repair or nucleotide excision repair pathways [17]; (iv) dysregulation of the cell cycle, such as *RB1* loss or cyclin E amplification [3,18]; and (v) epigenetic reprogramming affecting chromatin structure and gene expression [19]. Despite significant advances in next-generation sequencing (NGS) and molecular profiling, there is currently no validated genomic signature that predicts response to platinum in HG-SOC [20,21,22].

However, it is not possible to predict whether patients are sensitive, resistant or refractory, as a complete picture of the development of platinum resistance in HG-SOC patients is not yet available [3]. In particular, studies using next-generation sequencing (NGS) of tumors have enabled the identification of SNPs and/or gene signatures that can help stratify patients and predict prognosis, survival, or response to chemotherapy in various diseases such as breast, lung, ovarian, and gastric cancer [23,24,25]. Despite encouraging developments, no specific gene, signaling pathway, or signature is able to predict response to therapy in HG-SOC patients.

In this study, we performed targeted NGS of 409 cancer-associated genes in a cohort of HG-SOC patients stratified as platinum-sensitive, -resistant, or -refractory. Our aims were: (i) to identify mutations associated with specific platinum response categories, (ii) to assess their prognostic significance, and (iii) to investigate their translational potential as biomarkers and therapeutic targets. By characterizing the genomic landscape of platinum resistance, our results can support the identification of candidate biomarkers and inform precision oncology strategies for HG-SOC.

## 2. Materials and Methods

### 2.1. Patients’ Characteristics

We reviewed patients accrued at the Gynecologic Oncology Unit, Tommaso Campanella Foundation-UMG of Catanzaro. The study was conducted in accordance with the Declaration of Helsinki and approved by the “Pugliese-Ciaccio” institutional review board (IRB number: AOPC12404). In total, 24 tissue samples from patients with high-grade serous carcinoma (HG-SOC) were consecutively selected; all had undergone optimal upfront cytoreductive surgery. All patients received a standardized first-line chemotherapy regimen consisting of carboplatin and paclitaxel in combination with bevacizumab, with no alternative first-line regimens administered. Clinical data were collected retrospectively (Supplementary File S1). Written consent was obtained from all patients before processing their data at the time of hospitalization, even though the data did not include any personal identifying information. Whole blood and serum were collected before surgery for each patient.

The age range of the patients was 35–88 years with a median age of 62. Patients were classified by use of pathological surgical staging according to the 2009 International Federation of Gynecology and Obstetrics system (FIGO): stage I, 1 case; stage II, 2 cases; stage III, 21 cases. Patients diagnosed with high-grade HG-SOC were divided into three groups: chemotherapy-sensitive (*n* = 9), chemotherapy-resistant (*n* = 8), and chemotherapy refractory (*n* = 7), according to the results of the pre- or post-operative chemotherapy and the post-operative follow-up.

Group A, chemotherapy-sensitive group: patients with relapse at six months or more following the end of chemotherapy; group B, chemotherapy-resistant: patients with relapse within six months from the end of chemotherapy; group C, chemotherapy-refractory: patients who failed to achieve even partial response.

### 2.2. DNA Extraction and Quality Assessment

Genomic DNA was isolated from pathological tissue samples and peripheral blood using the GeneRead DNA FFPE Kit (Qiagen; Hilden, DE, Germany) and PureLink^®^ Genomic DNA Kit (Invitrogen, Carlsbad, CA, USA), respectively, in accordance with the manufacturer’s protocol. The concentration and integrity of the extracted DNA were evaluated using a Qubit Fluorometer (Invitrogen) and an Agilent 2200 TapeStation system (Agilent Technologies, Santa Clara, CA, USA). Samples of sufficient quality were used for library construction prior to sequencing.

DNA was extracted and sequenced from tumor samples (*n* = 24) and matched with normal peripheral blood lymphocytes (PBL) (*n* = 4) as controls. An average of 14 × 10^6^ reads per sample was generated. The mean sequencing depth was 760× (range: 288–2029), with a median gene uniformity of 85% (range: 60–96%). After filtering based on quality (≥30) and coverage (≥50), tumor samples showed an average of 2210.8 variants (range: 1169–6566), whereas peripheral blood leukocyte (PBL) samples exhibited a mean of 1190.8 variants (range: 1145–1236) (see Supplementary File S2).

Variant calling for single-nucleotide variants (SNVs) and small insertions/deletions (indels) was performed as described in the Section 2. Synonymous variants, along with those located in the 5′UTR, 3′UTR, intronic, intergenic (including upstream and downstream), and splice-region sequences, were excluded from further analysis. Somatic mutations were identified by subtracting variants detected in tumors from a “virtual normal pool” generated from the PBL samples as previously described [26,27,28,29]. Tumor-specific variants were further filtered out through the dbSNP141 and the 1000 Genomes Project databases. Non-synonymous variants were selected only if predicted to be damaging by both SIFT and Polyphen2 algorithms. Variants present in the COSMIC database were annotated as COSMIC.

### 2.3. Next-Generation Sequencing

Next-generation sequencing was performed using the Ion AmpliSeq™ Comprehensive Cancer Panel on the Ion Torrent platform (Thermo Fisher Scientific, Waltham, MA, USA), targeting all exons of 409 cancer-related genes. Library preparation was carried out with the Ion AmpliSeq Library Kit 2.0 using 40 ng of genomic DNA (quantified by Qubit 2.0 Fluorometer). Template preparation and emulsion PCR were conducted with the Ion PI™ OT2 kit v2 on the Ion OneTouch™ 2 system. Sequencing was performed using the Ion PI™ Sequencing 200 Kit v3 on an Ion PI™ v2 chip with the Ion Proton™ system.

Samples proceeded to sequencing only when the proportion of enriched Ion Sphere™ particles exceeded 70%. Up to four libraries were pooled per Ion PI™ v2 chip, and barcoding was performed using Ion Xpress™ Barcode Adapters (Thermo Fisher Scientific).

### 2.4. Bioinformatic Analysis

NGS data were processed using Torrent Suite software to generate reads, trim adapters, and exclude low-quality signals. Variants across the 16,000 amplicons targeting 409 genes were identified using Torrent Variant Caller (v5.0.3.5) within Torrent Suite v5.0.5 (Thermo Fisher Scientific). Variant filtering was based on coverage, quality, and allele frequency. For tumor samples, thresholds were set at coverage ≥ 50, quality score ≥ 30, and variant frequency ≥ 5%, while for peripheral blood leukocytes (PBL), coverage ≥ 10 and quality score ≥ 20 were applied. Sequencing metrics are provided in Supplementary File S2. To exclude germline variants, tumor variants were first compared against variants detected in pooled peripheral blood samples from a subset of patients (*n* = 4). Candidate somatic variants were then further filtered using dbSNP build 141 and the 1000 Genomes Project database. Annotation was performed against the COSMIC database, and the potential functional impact of variants was assessed using SIFT and PolyPhen-2 prediction tools [30,31]. The candidate variants were further filtered from gnomAD [32] and the European population from 1000 Genomes [33]. The potential deleterious effects of missense variants were evaluated using Rare Exome Variant Ensemble Learner (REVEL) [34], an ensemble prediction tool that integrates scores from 13 in silico algorithms (MutPred, FATHMM v2.3, VEST 3.0, PolyPhen-2, SIFT, PROVEAN, MutationAssessor, MutationTaster, LRT, GERP++, SiPhy, phyloP, and phastCons). REVEL scores range from 0 to 1, with higher scores indicating a greater likelihood of pathogenicity. Variant classification was performed according to oncogenicity criteria adapted from Horak et al. [35] and detailed in Supplementary File S3.

### 2.5. MSI Analysis

To assess MSI status, we used Easy-PGX ready MSI (Diatech Pharmacogenetics, Jesi, Italy). Tumor DNA was analyzed for 8 standardized microsatellite markers (*BAT25*, *BAT 26*, *NR21*, *NR22*, *NR24*, *NR27*, *CAT25*, *MONO27*).

According to the revised Bethesda guidelines, samples were classified as microsatellite stable (MSS) when no length alterations were observed, MSI-low (MSI-L) when one microsatellite showed instability, and MSI-high (MSI-H) when two or more microsatellites were altered [36].

### 2.6. Univariate and Multivariate Analyses

Univariate Cox regression analysis was performed to assess the association between mutated genes and clinical outcome. Genes resulted predictors of outcome in univariate analysis at *p*-value < 0.05, and together with the clinical–pathological covariates available, were used to perform multivariate survival analysis using a Cox’s multiple linear regression model, obtaining the best model using a stepwise approach (top-down selection).

### 2.7. TCGA Data Extraction and Survival Analysis

For external validation, we analyzed the TCGA ovarian cancer cohort (TCGA-OV) obtained from the Genomic Data Commons (GDC) data portal (updated August 2025), accessed through the cBioPortal platform [37]. Patients with available somatic mutation data were included (*n* = 405).

*BRCA1* and *BRCA2* mutation status was used to stratify patients, given their established association with platinum sensitivity. *BRCA1/2*-mutated patients were analyzed as a separate group. From the remaining *BRCA1/2* wild-type population, patients harboring at least one mutation in the predefined 12-gene panel identified in our cohort were classified as panel-positive. These genes were selected based on their recurrence and potential association with platinum resistance in our dataset. Gene mutation frequencies for the selected panel were calculated in both the overall TCGA cohort and the *BRCA1/2* wild-type subset, and are reported in Supplementary File S4. In cases where patients (*n* = 4) harbored both *BRCA1/2* mutations and mutations in the selected gene panel, they were assigned to the *BRCA1/2*-mutated group to ensure mutually exclusive classification.

The final groups were defined as follows: *BRCA1/2*-mutated patients (*n* = 28); *BRCA1/2* wild-type panel-positive patients (*n* = 37); *BRCA1/2* wild-type panel-negative patients (*n* = 336).

### 2.8. Statistical Analysis

Non-parametric statistical tests were applied due to the limited sample size. Con-tinuous variables were analyzed using Mann–Whitney U test or Kruskal–Wallis test, while categorical variables were analyzed using Fisher’s exact test. Correlation among categorical variables were further assessed using the χ^2^ test of independence. Standardized Pearson residuals were computed to quantify cell-wise deviations between observed and expected frequencies under the null hypothesis, with residuals exceeding |r| > 2 considered indicative of meaningful contributions to the χ^2^ statistic.

Overall survival (OS) was estimated using the Kaplan–Meier method, and difference between groups was evaluated using the log-rank (Mantel–Cox) test. Survival analyses for individual genes were performed independently using Graph-Pad Prism software 10.3.1. Hazard ratios (HRs) and 95% confidence intervals (CIs) were calculated using the log-rank (Mantel–Cox) method implemented in the software.

Multivariate survival analysis was performed using a Cox proportional hazards regression model to evaluate the independent prognostic impact of selected variables.

## 3. Results

### 3.1. Demographic Characteristics of the Patients Included in This Study

**Figure 1:**
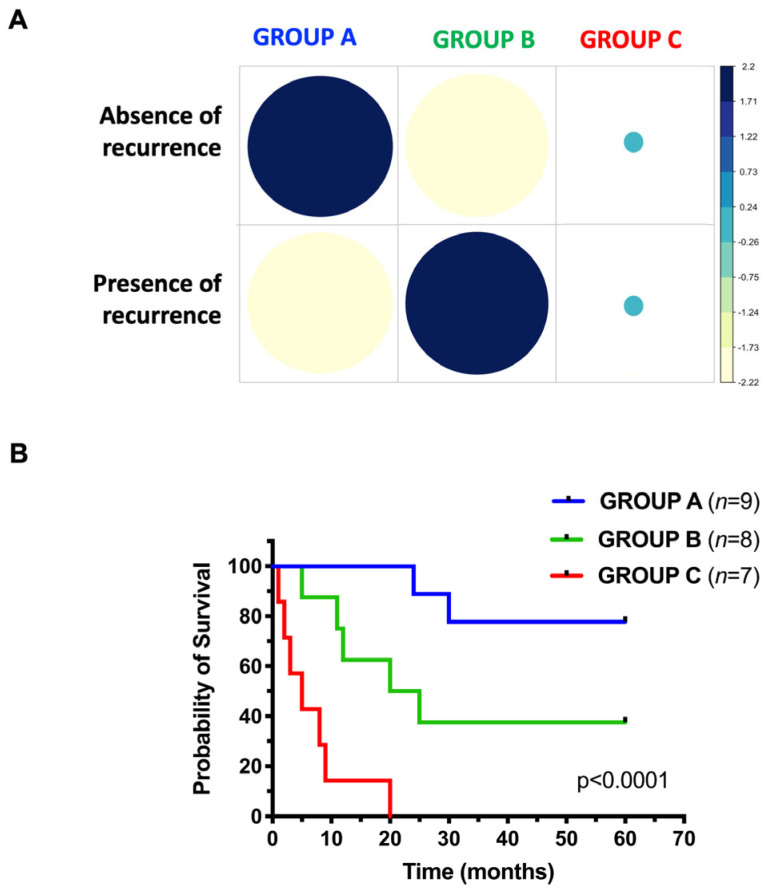
Enrichment of recurrent tumors in each response-to-therapy group. (**A**) Heatmap of standardized Pearson residuals derived from a χ^2^ test of independence, showing the correlation between recurrence status (rows) and chemotherapy response groups (columns: group A, sensitive; group B, resistant; group C, refractory). Residuals represent deviations between observed and expected frequencies under the null hypothesis. Circle size is proportional to the magnitude of the residuals. Blue and yellow colors indicate positive and negative residuals, respectively. (**B**) Kaplan–Meier curve showing the overall survival (OS) of patients enrolled in this study. Blue curve: group A; green curve: group B; red curve: group C.

The aim of this study was to determine the genetic determinants of response to platinum-based chemotherapy in patients with high-grade serous ovarian cancer (HG-SOC). To this end, we analyzed the mutation profiles of 24 HG-SOC patients representative of three different chemotherapy response patterns: group A, patients without recurrence (sensitive; *n* = 9); group B, patients with recurrence (resistant; *n* = 8); group C, patients with disease progression since initiation of therapy (refractory; *n* = 7). Figure 1A shows the correlation between recurrence status and chemotherapy response groups using standardized Pearson residuals from a χ^2^ test of independence. Patients in group A were enriched for absence of recurrence, whereas group B showed enrichment for recurrence. In contrast, group C did not display a clear enrichment pattern, with residuals close to zero, likely reflecting the intrinsic refractory nature of these patients, who typically exhibit disease progression from the start of therapy. These results validate the clinical stratification of patients based on treatment response.

The demographic characteristics of the patients included in this study are summarized in Table 1 and Supplementary File S1. The clinical outcomes of the included patients are shown in the Kaplan–Meier curves in Figure 1B. The 5-year survival rates were 77.8% in sensitive patients in group A (seven out of nine patients survived), 37.5% in resistant patients in group B (three out of eight patients survived) and 0% (zero out of seven patients survived) in refractory patients in group C. An apparent difference in survival rates was observed between all three groups, although interpretation is limited by sample size: all patients in group A, half of the patients in group B, and none of the patients in group C were still alive 20 months after diagnosis.

### 3.2. Known Genetic Determinants of Platinum Resistance

NGS analysis revealed heterozygous pathogenic mutations in *BRCA1/2* in six patients of group A and four in group B. Notably, no *BRCA1/2* mutations were found in refractory patients (Supplementary File S5). Microsatellite instability (MSI) was detected in three group A patients, with two showing high-level instability (see Supplementary Figures S1–S3 for curves). Of note, patient 2A had a missense mutation in *PMS1* (G501R), while 3A had a mutation in *ERCC5 (M679V).* MSI was absent in groups B and C. These findings suggest a potential association between *BRCA* mutations and MSI with improved chemotherapy response.

Moreover, we analyzed the expression of genes relevant to platinum sensitivity and resistance. Real-time PCR was used to assess the expression level of the copper transporter *CTR1* [38] and the efflux pumps *ATP7A/7B* [39], while quantitative PCR was employed to determine the copy number (CN) of *CCNE1* [40] and *RB1* [18,41]. In tumor tissues, no changes or detectable mRNA expression levels were observed for the *ATP7* copper transporters or the *CTR1* transporter. Similarly, no significant differences were detected among the three groups in genes involved in the proliferative RB1 signaling pathway. Specifically, the median *RB1* CN was 1.9 in group A, 1.6 in group B, and 1.8 in group C patients (Supplementary Figure S4A). Notably, significant *RB1* gene loss was observed only in two patients from group B (7B and 13B). In contrast, the median CN value of cyclin E was higher in group A patients (4.8) compared with group B (2.5) and group C (1.9) patients (Supplementary Figure S4B). Although these differences did not reach statistical significance, increased cyclin E gene copy numbers were detected in four of six group A patients (1A, 3A, 7A, and 13A), in three group B patients (7B, 9B, and 12B), and in only one group C patient (7C).

Overall, these findings do not provide a clear mechanistic explanation for treatment resistance. Therefore, we proceeded with next-generation sequencing analyses to further investigate the potential molecular determinants underlying resistance in our patient cohort.

### 3.3. NGS

We performed the targeted sequencing of HG-SOC with the amplicon technology of Ion Torrent Platform using the Comprehensive Cancer Panel, which provides complete whole exon coverage of the 409 most important cancer-associated genes. See Section 2 for details.

In total, 1367 putative protein-altering somatic variants were identified (Supplementary File S5), affecting 301 genes. The number of variants per tumor ranged from 16 to 348, with a median of 32. A total of 25 COSMIC-annotated variants were detected (Supplementary File S5), corresponding to a median of one variant per tumor (range: 1–3). The median number of mutated genes per tumor was 32 (range: 13–169).

### 3.4. Genes Mutated in All Three Response-to-Therapy Groups

**Figure 2:**
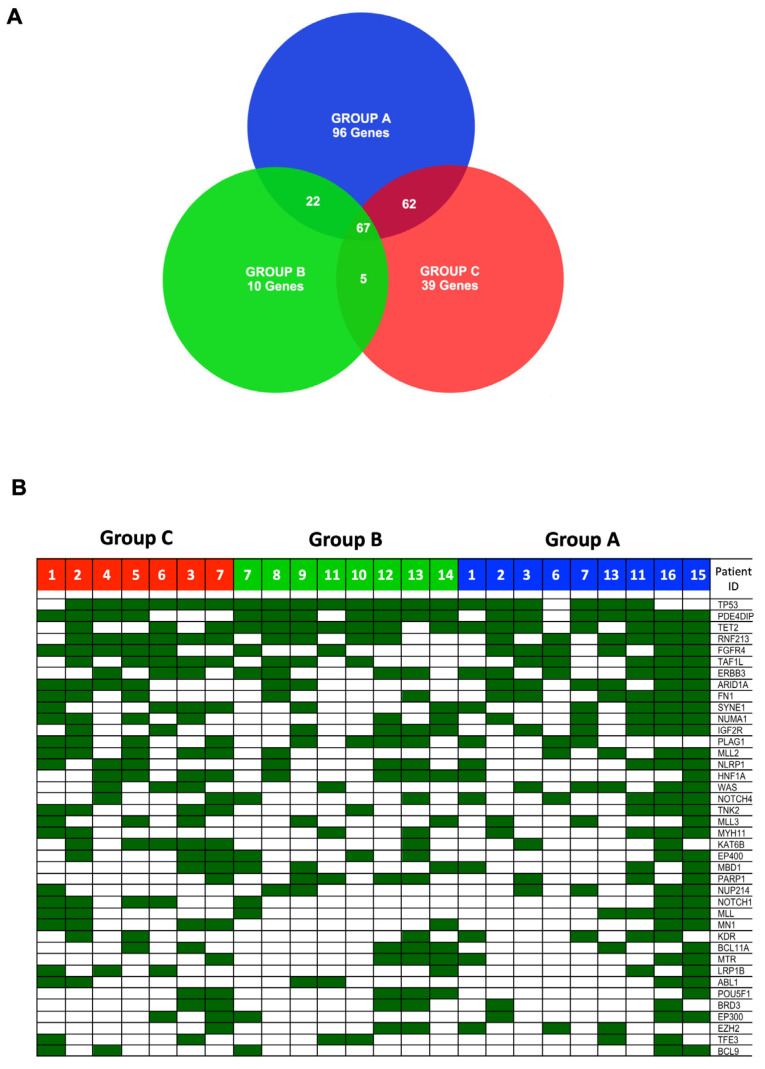
Distribution of mutated genes in patients with high-grade serous ovarian cancer (HG-SOC). (**A**) Venn diagram showing the number of genes mutated exclusively in a single group or shared by two or all three patient groups. (**B**) Heat map illustrating the distribution of mutated genes across the different response-to-therapy groups. Columns represent the 24 HG-SOC patients included in the study, grouped by therapy response, while rows represent mutated genes.

Subsequently, we compared the lists of genes specifically mutated in patients from groups A, B, or C (Figure 2A and Supplementary File S5). A total of 247 genes were mutated in group A, 104 genes in group B, and 173 genes in group C (Supplementary File S5, sheets group A, B and C, respectively). The number of genes mutated in at least one patient within each response-to-therapy group was 67 (Figure 2A and Supplementary File S5, sheet Common).

As expected, the most frequently mutated gene in HG-SOC was *TP53* (83%). Mutations in *TP53* were observed in 100% (8/8) of the resistant group B, 86% (6/7) of refractory group C and 67% (6/9) of sensitive group A patients (Table 2 and Figure 2B). We identified 19 different mutations in the *TP53* gene: one gain-of-function, eight loss-of-function, and 10 unclassified. All variants were localized in the DNA-binding domain (residues 94–292) of *TP53*, except for one present in the N-terminal proline-rich domain (R89G). See Supplementary Figure S5A.

Frequent mutations in HG-SOC across all response-to-therapy groups were also identified in Phosphodiesterase 4D Interacting Protein *(PDE4DIP*) (75%), Ten-eleven translocation 2 (*TET2*) (71%), Ring Finger Protein 213 (*RNF213)* (67%), Fibroblast Growth Factor Receptor 4 (*FGFR4*) (54%), TATA-box binding protein associated factor 1 like (*TAF1L*) (54%), Erb-b2 receptor tyrosine kinase 3 (*ERBB3*) (54%) (Table 2 and Supplementary Figure S5).

Notably, we found that AT-rich interacting domain-containing protein 1A gene (*ARID1A*) was mutated in 12 HG-SOC patients (50%). *ARID1A* variants were identified in six group A (67%), two group B (25%) and four group C (57%) patients (Table 2).

In our cohort, most patients (nine, 38%) presented the cancer-associated frameshift mutation Q1142fs (COSM251400). The remaining 3 patients in group A patients carried low allelic frequency mutations (Supplementary File S5 and Figure S5B). *ARID1A* mutations frequently co-occur with alterations in genes encoding PI3K pathway components. In our cohort, six of 12 patients with *ARID1A* mutations had defects in the PI3K/AKT pathway. We found the *PIK3CA* E545K mutation in one group C patient (1C). Two refractory patients (2C and 4C) carried the D292N mutation of *AKT1* and the G287S mutation of *AKT2*, respectively. A pathogenic *TSC1* (R509*) mutation was found in a group A patient (7A), while a *TSC2* mutation (D1406Y) was present in a group B patient (9B).

Supplementary Figure S6 shows representative Sanger sequencing analysis of SNVs identified in the manuscript as validation.

### 3.5. Genes Mutated Exclusively Within Specific Response-to-Therapy Groups

However, we were interested in identifying genes specifically mutated in the resistant or refractory patients groups. We found that 96 genes were mutated exclusively in group A (Supplementary File S5, sheet Ex_A), 10 genes were mutated exclusively in group B (Supplementary File S5, sheet Ex_B), and 39 genes were mutated exclusively in group C (Supplementary File S5, sheet Ex_C) (Figure 2A).

In addition, 22 mutated genes were found to be common between group A and group B patients, 62 mutated genes were common between group A and group C, and five mutated genes (*ATF1, MLLT10, FANCC, NRAS,* and *TOP1*) were common between group B and group C (Supplementary File S5, sheets A-B, A-C, B-C, respectively). We referred to Horak et al. [35] for a more robust identification of oncogenic alterations and accordingly applied the REVEL score, an ensemble predictor integrating multiple in silico tools (see Section 2 for details), to optimize overall predictive performance. Based on these criteria, the variants identified in this study were classified as oncogenic (O), likely oncogenic (LO), variants of uncertain significance (VUS), likely benign (LB), or benign (B).

Given the limited cohort size, the observed associations should be considered exploratory and hypothesis-generating. Larger, independent studies are warranted to establish whether these alterations represent reproducible predictors of platinum response and/or survival outcomes.

**Figure 3:**
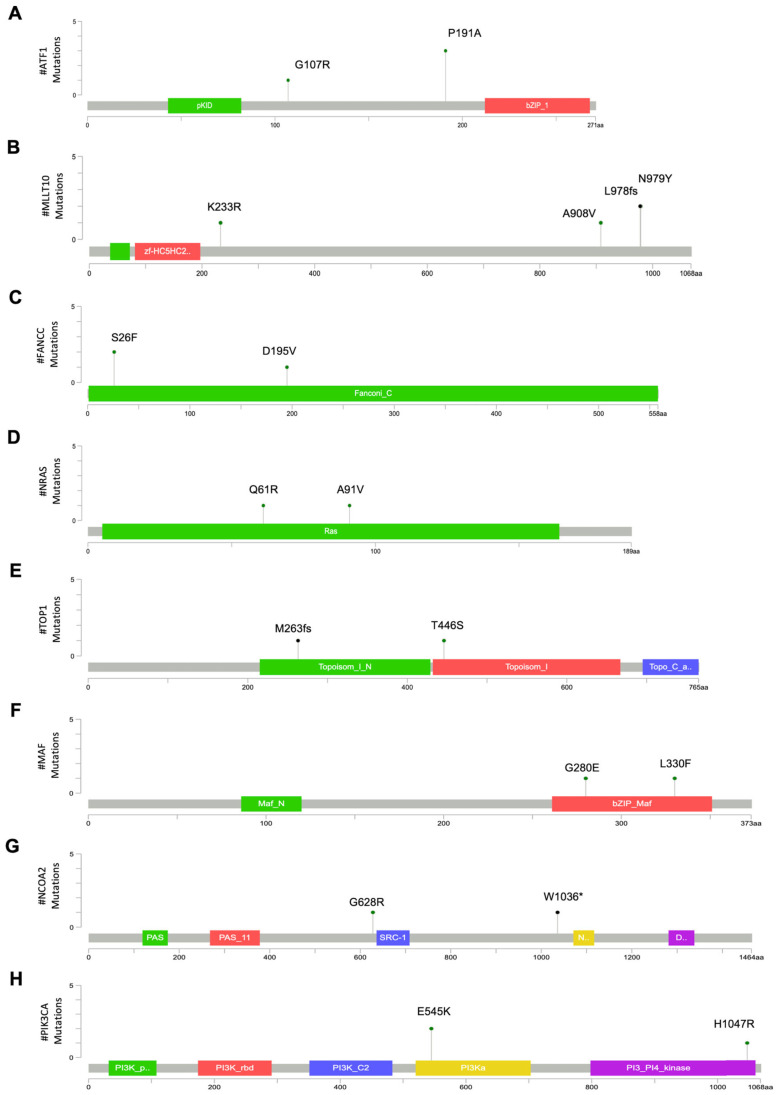
Mutation mapper plots showing the positions of amino acid changes in genes mutated within specific response-to-therapy groups. (**A**–**E**). Mutation mapper plots showing the variants identified in genes mutated in both group B and group C patients: *ATF1* (**A**), *MLLT10* (**B**), *FANCC* (**C**), *NRAS* (**D**), and *TOP1* (**E**). (**F**–**H**). Mutation mapper plots showing the variants identified in genes exclusively mutated in group C patients: *MAF* (**F**), *NCOA2* (**G**), and *PIK3CA* (**H**). Mutation mapper plots were created with: https://www.cbioportal.org/mutation_mapper on 8 April 2026. * stop codon.

For genes common to groups B and C, *ATF1* is a leucine zipper transcription factor that binds to cAMP-inducible promoters. This gene was mutated in four patients, two in group B (25%), and two in group C (28%). Three patients carried the P191A mutation, which is located within the Q2 domain (a glutamine-rich constitutive activation domain). Based on its relatively high allele frequency in population databases (gnomAD ~3%), this variant was classified as likely benign (LB). The other refractory patient carried the G107R missense mutation, which falls within a linker domain (Table 3 and Figure 3A).

*MLLT10*, a cofactor of histone methyltransferase, was mutated in one resistant (12,5%, 10B) and two refractory patients (29%, 1C and 3C). Patients 10B and 3C presented a combination of a putatively oncogenic frameshift (L978fs) followed by a missense mutation (N979Y) (Table 3 and Figure 3B).

*FANCC* was mutated in three patients from group B (38%) and one patient of group C (14%). Two resistant patients carried the S26F mutation, while the third had the D195V mutation; the group C patient presented with a variant at the splice acceptor site (Figure 3C). The S26F and D195V mutations were classified as VUS, whereas the splice acceptor site mutation was likely oncogenic (Table 3).

*NRAS* was mutated in one resistant patient (12,5%, Q61R) and one refractory patient (14%, A91V). Q61R is a well-established hotspot oncogenic mutation [42], whereas A91V, although reported in several cancer types [43], has not yet been functionally characterized (Figure 3D).

For the *TOP1* gene, one patient in group B carried a missense mutation (T446S), while one patient in group C had a frameshift deletion (M263fs) leading to a premature stop codon; both variants were classified as VUS (Figure 3E and Table 3).

Each of the 10 genes that were exclusively mutated in group B (*UGT1A1, BLNK, FOXO1, CDH2, SF3B1, DDIT3, CDH5, BAI3, POT1, MRE11A*) was mutated in only one patient (Supplementary File S5, Ex_B).

In group C, a total of 39 genes were mutated (Supplementary File S5, Ex_C), of which nine were mutated in at least two patients (*MAF, KIT, PIK3CA, NCOA2, BCL3, BCL10, FOXP4, HRAS, CEBPA*).

Notably, *MAF*, a transcription factor belonging to the *AP1* protein family, was mutated in two refractory (29%) group C patients (Table 3). The two identified mutations (G280E, L330F) are located in the leucine zipper domain of the basic region (bZIP), which mediates protein dimerization and DNA binding [44] (Figure 3F). Both mutations were predicted as VUS (Table 3).

The gene encoding the Nuclear Receptor Coactivator 2 (*NCOA2*) was mutated in two patients (29%) from group C (Table 3). Two distinct variants were identified: a nonsense mutation (W1036*) and a missense variant (G628R), the latter located in proximity to the steroid receptor coactivator (SRC) domain; both were classified as variants of uncertain significance (Table 3 and Figure 3G).

Two refractory patients in group C (29%) carried gain-of-function mutations of the gene encoding the catalytic subunit alpha of the Phosphatidylinositol-4,5-Bisphosphate 3-Kinase (*PIK3CA*), E545K, and H1047R, which were localized in the helix and kinase domains, respectively (Figure 3H). Both mutations widely described [45,46,47] and falling in hotspot domains of the gene were predicted to be oncogenic (Table 3).

### 3.6. Statistically Associated Mutations in Refractory Patients

In addition, by use of a X^2^ test, we identified four genes showing statistical association with group C in this exploratory analysis: *TET1, TAF1, KAT6B, FANCA* (Table 3 and Supplementary File S5).

**Figure 4:**
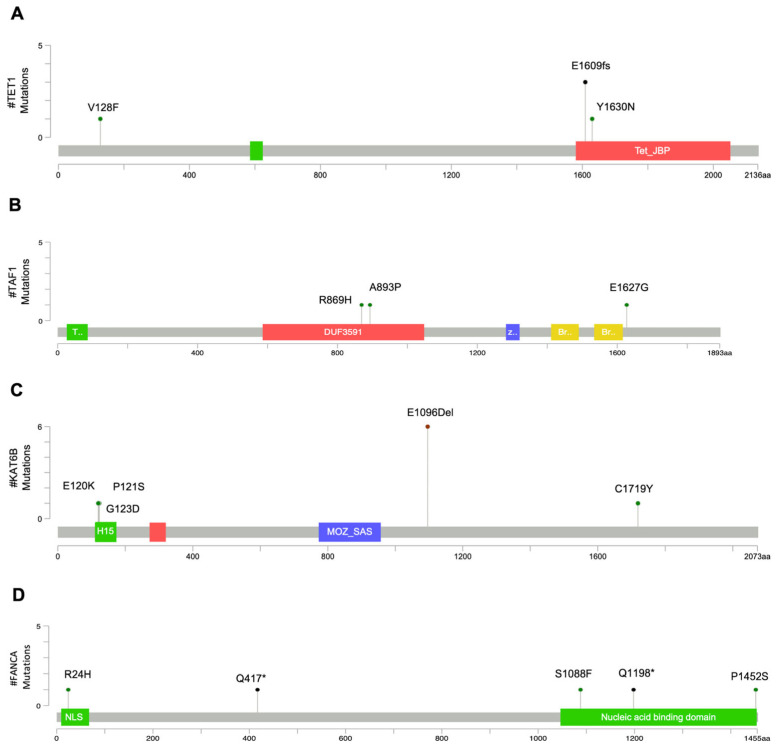
Mutation mapper plots of mutated genes associated with patients in group C. Mutation mapper plots show the positions of amino acid changes corresponding to the variants identified in: *TET1* (**A**), *TAF1* (**B**), *KAT6B* (**C**), and *FANCA* (**D**). Mutation mapper plots were created with: https://www.cbioportal.org/mutation_mapper on 08 April 2026. FANCA mutation mapper plot was modified according to Palovcak A et al. [49]. H15: histone H1/H5 like domain; NLS: nuclear localization signal; * stop codon.

As for group C, Ten-eleven translocated 1 (*TET1*) catalyzes the initial step in the DNA demethylation process [48]. *TET1* was mutated in four patients in group C (57%). Three patients had the E1609fs frameshift mutation, classified as oncogenic and located within the core catalytic domain (TET_JBP domain), resulting in a premature stop codon (Supplementary File S5). One of these patients (1C) also carried the VUS variant Y1630N. The remaining patient in group C carried the V128F variant, which was classified as likely benign. See Table 3 and Figure 4A for details.

TATA-box binding protein associated factor 1 (*TAF1*) belongs to the TFIID complex necessary for the initiation of transcription. *TAF1* was mutated in three patients from group C (43%) and in one patient from group A (11%). The refractory patients harbored three distinct missense variants (A839P, R869H, and E1627G, respectively). Although all variants were classified as of uncertain significance, the R869H substitution is located within the DUF3591 domain, a highly conserved region, suggesting potential biological relevance. See Table 3 and Figure 4B for details.

The Lysine Acetyltransferase 6B (*KAT6B*) gene, which encodes a histone acetyltransferase [50], was mutated in five patients from group C (71%; 2, 3, 5, 6, 7 C), two patients from group A (22%; 3 and 16 A) and one patient from group B (12,5%; 13 B). Six patients had the E1096 in-frame deletion located in the domain enriched with glutamate/aspartic acid residues, which is not predicted to affect the protein function and was therefore classified as benign. One patient (3C) carried four different substitutions, three of which (E120K, P121S, G123D) were located in the N-terminal H15 (Histone H1/H5 like) domain involved in nuclear translocation of the enzyme, while C1719Y was located in the C-terminal domain involved in transcriptional activation (Figure 4C). All four mutations were classified as variants of uncertain significance (Table 3).

Finally, the Fanconi anemia complementation group A (*FANCA*) gene, that encodes a DNA repair protein, was mutated in four patients from group C (57%) and one patient from group A (11%). Each *FANCA*-mutated patient in group C carried a different mutation: R24H within the *FANCG* binding and NLS domain; the truncating Q417* mutation; the S1088F, Q1198*, and P1452S mutations within the nucleic acid binding domain at the C-terminus (Figure 4D). The nonsense mutations, Q417* and Q1198*, were predicted loss-of-function mutations and classified as oncogenic (Table 3).

### 3.7. Clinical Significance of Mutated Genes

Subsequently, we investigated the potential clinical relevance of the genes associated with specific response-to-therapy groups through a survival analysis using the log-rank test. Given the limited sample size and multiple comparisons, these findings should be interpreted with caution and considered exploratory. In our HGSOC cohort, mutations in *FANCA*, *ATF1*, *MAF*, *NCOA2*, *PIK3CA*, and *TET1* were associated with shorter overall survival in exploratory analyses (Table 4 and Supplementary Figure S7). Although several genes showed nominally significant associations with overall survival, the corresponding hazard ratios displayed wide confidence intervals, reflecting the limited sample size and small number of mutation-positive cases.

The median survival of patients with mutations in these genes ranged from 2.5 to 9 months, whereas the median survival of patients without mutations ranged from 27.5 to 45 months. However, given the limited sample size and the inherent risk of overfitting, we sought independent confirmation in the TCGA-OV dataset.

We additionally queried the TCGA-OV cohort through cBioPortal and repeated mutation-group-based survival analyses in this larger, independent dataset.

To further confirm the relevant genes identified and previously described in our cohort, we analyzed the correlation with survival of a 12-gene panel consisting of: *ATF1*, *MLLT10*, *FANCC*, *NRAS*, *TOP1*, *TET1*, *TAF1*, *KAT6B*, *FANCA*, *MAF*, *NCOA2*, and *PIK3CA*.

**Figure 5:**
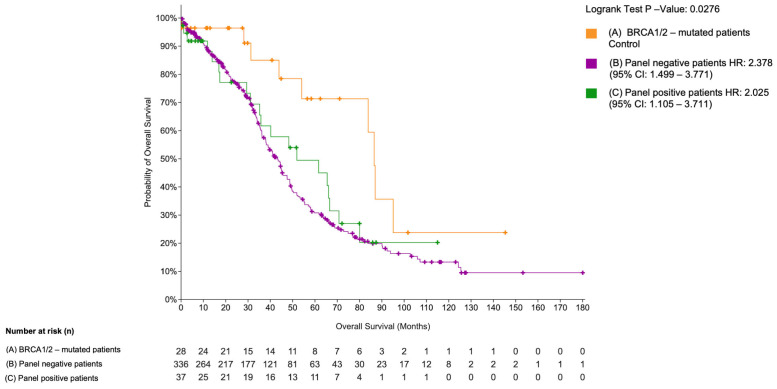
Overall survival in the TCGA ovarian serous carcinoma cohort stratified by mutational status. Kaplan–Meier overall survival analysis of TCGA-OV patients stratified into three groups: *BRCA1/2*-mutated patients (orange, *n* = 28), *BRCA1/2* wild-type panel-positive patients (green, *n* = 37), and *BRCA1/2* wild-type panel-negative patients (violet, *n* = 336). Panel-positive indicates the presence of a mutation in at least one gene of the 12-gene panel. The log-rank test *p*-value is shown. The number of patients at risk at each time point is reported below the plot. Figure was created with: https://www.cbioportal.org/comparison/survival?comparisonId=69d6e6714c68fa15d62ae660 accessed on 5 April 2026.

We performed a Kaplan–Meier analysis using the TCGA dataset for high-grade serous ovarian cancer [37]. Among 405 patients with available mutation data, 28 harbored *BRCA1/2* mutations and were analyzed separately due to their known association with platinum sensitivity. Among *BRCA1/2* wild-type patients, 37 cases (9.1%) carried mutations in at least one of the 12 genes identified in our cohort and were classified as BRCA1/2 wild-type panel-positive. These patients showed reduced overall survival compared to the group, including patients without *BRCA1/2* mutations and without alterations in the 12-gene panel (*n* = 336) (Figure 5).

A statistically significant difference in overall survival was observed across the three groups (log-rank *p* = 0.0276), despite the relatively small number of patients in the BRCA-mutated group. Carriers of *BRCA1/2* mutations had the most favorable overall survival, consistent with their recognized sensitivity to platinum-based chemotherapy and PARP inhibition [8,51,52]. In contrast, patients with mutations in the 12-gene group showed significantly poorer survival (HR = 2.025, 95% CI: 1.105–3.711), indicating a more aggressive disease course. Overall, these results support the notion that alterations in novel susceptibility genes may identify patients with a particularly unfavorable prognosis who may require alternative therapeutic approaches.

We then performed a multivariate Cox regression analysis to assess whether the genes associated with poor outcome in univariate analyses remained independent predictors when adjusted for clinical–pathological variables. Mutations in *FANCA* and *ATF1* emerged as potential independent predictors of poor survival, conferring a 21.4-fold and 15.2-fold increased risk of death, respectively (Table 5). Peritoneal carcinomatosis was also identified as an independent adverse prognostic factor (HR = 5.14). Patients with peritoneal carcinomatosis had a median survival of 9.4 months compared with 38 months in those without peritoneal involvement.

## 4. Discussion

In this exploratory study, we investigated genomic alterations associated with differential response to platinum-based chemotherapy in high-grade serous ovarian carcinoma (HG-SOC), with particular emphasis on platinum-resistant and platinum-refractory disease. By integrating targeted next-generation sequencing of a clinically well-annotated cohort with independent validation in the TCGA-OV dataset, we identified a set of genomic alterations associated with platinum resistance and adverse clinical outcome. Our findings extend current knowledge by highlighting the contribution of transcriptional and epigenetic dysregulation, alongside non-canonical DNA repair defects, to aggressive HG-SOC phenotypes.

Consistent with previous large-scale genomic analyses, *TP53* mutations were ubiquitous across all platinum-response groups, confirming their central but non-discriminatory role in HG-SOC biology [53]. Likewise, *BRCA1/2* mutations and other markers of homologous recombination deficiency (HRD) were predominantly observed in platinum-sensitive patients, in line with extensive evidence linking HRD to improved platinum and PARP inhibitor response [54,55,56]. In contrast, alterations in classical resistance mechanisms such as RB1 loss, cyclin E amplification, or drug efflux pathways did not adequately explain the refractory phenotype in our cohort, reflecting the limited ability of established markers to capture intrinsic platinum resistance [57,58].

A central observation of this study is the enrichment of oncogenic alterations affecting transcriptional regulation, epigenetic modulation, DNA repair, and oncogenic signaling in platinum-resistant and platinum-refractory tumors. Although individual mutations were often infrequent, they converged on biologically coherent pathways, supporting emerging models in which therapy resistance arises from network-level dysregulation rather than single driver events [59]. This concept is increasingly recognized across solid tumors, including ovarian cancer, where transcriptional plasticity and chromatin remodeling enable adaptive responses to chemotherapy-induced stress [60,61].

Among transcriptional regulators, *ATF1* and *MAF* emerged as notable candidates. ATF1 is a cAMP-responsive transcription factor implicated in oncogenic transcriptional programs and has been shown to interact with *BRCA1* and DNA damage response machinery [62,63]. In our cohort, *ATF1* mutations were associated with significantly reduced survival and retained independent prognostic value in multivariate analysis. Although functional validation is required, alterations in *ATF1* may disrupt stress-response transcriptional programs critical for platinum-induced apoptosis. Similarly, *MAF*, a member of the AP-1 transcription factor family, has been implicated in therapy resistance and metastatic progression in several malignancies [64,65]. Mutations affecting its DNA-binding or dimerization domains may impair apoptotic signaling and promote survival under cytotoxic pressure.

Epigenetic regulators constituted another prominent class of altered genes in platinum-refractory disease. Epigenetic reprogramming has been increasingly recognized as a driver of chemotherapy resistance, enabling reversible phenotypic adaptation without reliance on genetic selection [61,66,67]. In ovarian cancer, this plasticity has been linked to stem-like states and poor response to platinum therapy [68,69], supporting the relevance of our findings. Oncogenic variants in *TET1*, *KAT6B*, *TAF1*, and *MLLT10* suggest a widespread disruption of chromatin accessibility and transcriptional control. In particular, loss-of-function alterations in *TET1*, a key regulator of DNA demethylation, may contribute to aberrant methylation patterns and transcriptional silencing. Notably, *TET* activity is influenced by cellular redox status, linking oxidative stress to epigenetic regulation and potentially to chemotherapy response [70]. Similarly, *KAT6B*, a histone acetyltransferase, plays a central role in regulating chromatin accessibility and gene expression, and its dysregulation may promote oncogenic transcriptional programs. Emerging evidence suggests that targeting epigenetic modifiers, including histone acetylation pathways, may represent a therapeutic opportunity in ovarian cancer [71]. Alterations in transcription-associated regulators such as *TAF1* and *MLLT10* further support the presence of coordinated transcriptional rewiring in resistant tumors. In particular, *TAF1*, a core component of the transcription initiation machinery, has been identified as a recurrently altered driver in aggressive and therapy-resistant metastatic tumors [72], where it is associated with widespread transcriptional and signaling reprogramming. Importantly, these mechanisms are potentially targetable, as epigenetic therapies are being explored to restore chemosensitivity and improve treatment outcomes [73].

Alterations in DNA repair pathways beyond *BRCA1/2* also proved clinically significant. Mutations in *FANCA* and *FANCC*, key components of the Fanconi anemia pathway, were enriched in resistant and refractory tumors and associated with poorer survival. While homologous recombination deficiency is generally linked to platinum sensitivity, partial or aberrant disruption of DNA repair pathways may instead promote genomic instability and tumor heterogeneity, facilitating adaptive resistance [74,75]. The independent prognostic value of *FANCA* mutations observed in our cohort highlights the complexity of DNA repair alterations in HG-SOC and cautions against simplistic interpretation of repair defects as uniformly predictive of treatment sensitivity.

Consistent with this complexity, we identified mutations in oncogenic signaling pathways, including *PIK3CA* and *NRAS,* exclusively in resistant or refractory patients. Activation of PI3K/AKT signaling has been repeatedly implicated in platinum resistance through enhanced survival signaling and impaired apoptosis [76,77]. Although *NRAS* mutations are uncommon in HG-SOC, MAPK pathway activation has been associated with adaptive resistance mechanisms and may represent a targetable vulnerability in selected patients [78].

Importantly, the possible prognostic relevance of these alterations was supported by independent validation in the TCGA-OV cohort. Patients harboring oncogenic variants in a 12-gene panel derived from our study exhibited significantly worse survival compared with both the overall cohort and *BRCA1/2*-mutated cases. This external validation supports our findings and aligns with recent efforts to refine molecular stratification of HG-SOC beyond HRD status alone. Notably, our gene panel captures pathways associated with transcriptional and epigenetic control rather than classical genomic instability, potentially identifying a subgroup of patients with intrinsically aggressive disease biology.

From a translational perspective, the identification of alterations affecting transcriptional regulation, epigenetic plasticity, and DNA repair pathways may open new therapeutic opportunities for platinum-resistant HG-SOC. Among emerging strategies, proteolysis-targeting chimeras (PROTACs) have gained attention as a modality for selective protein degradation [79,80], enabling the targeting of proteins, including transcription factors and chromatin regulators, often considered “undruggable” [81,82]. In parallel, rational combination strategies are being explored to overcome adaptive resistance [83,84,85]. These insights may help guide future patient stratification and mechanism-based therapeutic approaches.

Some limitations must be acknowledged. Some variants observed in multiple patients (e.g., *KAT6B* E1096del, *ATF1* P191A) are likely to represent recurrent germline variants rather than tumor-specific events. This underscores that, particularly in small cohorts, recurrence should be interpreted cautiously and in the context of population-level data. Given the modest cohort size, this study should be regarded as hypothesis-generating. In addition, subgroup-based survival analyses were performed on very small patient groups, which limits statistical power and increases the risk of false positive findings. The consistency of pathway-level enrichment and the independent validation in the TCGA-OV cohort partially mitigate this limitation, but prospective validation in larger series is required. Multiple testing correction was not applied due to the hypothesis-generating nature of the study, and functional characterization of individual variants remains necessary. Nonetheless, the convergence of pathway-level enrichment, consistent survival associations, and independent TCGA validation supports the biological plausibility and clinical relevance of the identified genomic signature.

Overall, our findings suggest that platinum resistance in HG-SOC is driven by complex alterations affecting transcriptional regulation, epigenetic plasticity, DNA repair, and oncogenic signaling, rather than by single canonical resistance mechanisms. These results support the rationale for extending genomic profiling beyond *BRCA1/2* status to better capture biological heterogeneity and clinical risk in HG-SOC.

## 5. Conclusions

In conclusion, this exploratory study identifies a set of genomic alterations converging on transcriptional regulation, epigenetic modification, DNA repair, and oncogenic signaling that are associated with platinum resistance and poor prognosis in high-grade serous ovarian carcinoma. Independent validation in the TCGA ovarian cancer cohort supports the clinical relevance of a multi-gene mutational signature that distinguishes patients with aggressive disease biology from *BRCA1/2*-mutated cases. These findings suggest that expanding genomic profiling beyond BRCA status may improve patient stratification and help identify individuals at higher risk of early relapse or treatment failure.

However, given the limited sample size, the identified associations require confirmation in larger, prospectively collected cohorts, as well as functional studies to clarify the biological mechanisms underlying platinum resistance and to assess their potential as therapeutic targets.

## Figures and Tables

**Table 1:** Demographics information of HG-SOC patients (*n* = 24).

Clinical Characteristics		
**Mean age (years range)**	62 (35–88)	
**Histology**	HG-SOC	
	**Patients**	
**Stage**	**No**	**%**
**I**	1	4.2
**II**	2	8.4
**III**	21	87.5
**Response to therapy**	**No**	**%**
Sensitive	9	37.5
Resistant	8	33.3
Refractory	7	29.2
**Peritoneal carcinomatosis**	**No**	**%**
Presence	11	45.8
Absence	13	54.2
**Ascitic fluid**	**No**	**%**
Presence	16	66.7
Absence	8	33.3
**Mean tumor diameter (mm range)**	98.6 (60.5–228.5)	
**Mean OS (months range)**	29.25 (range 1–60)	

**Table 2:** The most frequently mutated genes. The table contains the most frequently mutated genes in the HG-SOC cohort (all patients *n* = 24) and in the different groups of response to therapy (sensitive, group A, *n* = 9; resistant, group B, *n* = 8; refractory, group C, *n* = 7). Gene symbols are reported according to HGNC nomenclature. Full gene names are provided in Supplementary File S3.

Gene Symbols	All Patients (Mean%)	Group A (%)	Group B (%)	Group C (%)
*TP53*	84	67	100	86
*PDE4DIP*	75	78	88	57
*TET2*	70	78	88	43
*RNF213*	68	67	50	86
*FGFR4*	54	67	25	71
*TAF1L*	54	56	38	71
*ERBB3*	54	67	50	43
*ARID1A*	50	67	25	57
*FN1*	45	67	25	43
*SYNE1*	46	56	25	57
*NUMA1*	46	56	25	57
*IGF2R*	46	56	50	29
*PLAG1*	46	33	50	57
*MLL2*	42	44	12	71
*NLRP1*	42	44	38	43
*HNF1A*	43	22	50	57
*WAS*	37	56	12	43
*NOTCH4*	37	56	25	29
*TNK2*	33	33	12	57
*MLL3*	33	33	25	43
*MYH11*	33	44	25	29
*KAT6B*	33	22	12	71
*EP400*	33	22	38	43
*MBD1*	33	33	38	29
*PARP1*	32	33	50	14
*BCL11A*	29	22	38	29
*NOTCH1*	29	22	12	57
*MN1*	29	22	12	57

**Table 3:** Genes associated with response to therapy. The table lists the genes that are most frequently mutated in one of the different groups of response to therapy. For each variant of the allele frequency in population databases (gnomAD, 1000 genomes), the pathogenicity prediction score (REVEL), the oncogenicity criteria adapted from Horak et al., and the final classification are reported. Abbreviations: gnomAD, Genome Aggregation Database; B, benign; LB, likely benign; O, oncogenic; LO, likely oncogenic; VUS, variant of uncertain significance; N/A, not available; * stop codon.

	Gene ID	Chr	Sample Name	Mutation	REVEL Score	gnomAD	1000 Genomes	Oncogenic Criteria	Classification
**GROUP B-C**	*ATF1*	chr12	6C	G107R	0.624	0.00003	N/A	OP1, OP4	**VUS**
	*ATF1*	chr12	8B, 9B, 4C	P191A	0.225	0.03000	0.0091853	SBS1	**LB**
	*MLLT10*	chr10	1C	A908V	0.087	N/A	N/A	OP4, SBP1	**VUS**
	*MLLT10*	chr10	3C	K233R	0.138	0.00050	0.0011981	OP4	**VUS**
	*MLLT10*	chr10	10B, 3C	N979Y	0.085	N/A	N/A	OP4, SBP1	**VUS**
	*MLLT10*	chr10	10B, 3C	L978fs	N/A	N/A	N/A	OP4	**VUS**
	*FANCC*	chr9	2C	c.1330-1 C>T	N/A	N/A	N/A	OVS1, OP4	**LO**
	*FANCC*	chr9	9B, 13B	S26F	0.230	0.00579	0.0030000	OP4	**VUS**
	*FANCC*	chr9	7B	D195V	0.417	0.00394	0.003	OP4	**VUS**
	*NRAS*	chr1	3C	A91V	0.301	0.00001	N/A	OM1, OP4	**VUS**
	*NRAS*	chr1	12B	Q61R	0.888	N/A	N/A	OS1, OS2, OS3, OP1, OP4	**O**
	*TOP1*	chr20	7B	T446S	0.304	N/A	N/A	OM1, OP4	**VUS**
	*TOP1*	chr20	2C	M263fs	N/A	N/A	N/A	OM1, OP4	**VUS**
**GROUP C Exclusive**	*MAF*	chr16	4C	L330F	0.893	0.0000012	N/A	OM1, OP1, OP4	**VUS**
	*MAF*	chr16	1C	G280E	0.707	0.0000016	N/A	OM1, OP1, OP4	**VUS**
	*NCOA2*	chr8	3C	G628R	0.148	0.0008770	N/A	OP4	**VUS**
	*NCOA2*	chr8	1C	W1036 *	N/A	N/A	N/A	OM1, OP4	**VUS**
	*PIK3CA*	chr3	1C	E545K	0.654	N/A	N/A	OS1, OS2, OM1, OP3	**O**
	*PIK3CA*	chr3	7C	H1047R	0.455	N/A	N/A	OS1, OS2, OM1, OP3, OP4	**O**
**GROUP C Associated**	*TET1*	chr10	5C	V128F	0.03	0.0162000	0.0050000	SBS1, SBP1	**LB**
	*TET1*	chr10	1, 3, 6 C	E1609fs	N/A	N/A	N/A	OVS1, OM1, OP4	**O**
	*TET1*	chr10	1C	Y1630N	0.157	N/A	N/A	OM1, OP4	**VUS**
	*TAF1*	chrX	2C	A893P	0.366	N/A	N/A	OM1, OP4	**VUS**
	*TAF1*	chrX	3C	E1627G	0.264	N/A	N/A	OM1, OP4	**VUS**
	*TAF1*	chrX	5C	R869H	0.617	N/A	N/A	OM1, OP1, OP4	**VUS**
	*KAT6B*	chr10	3C	P121S	0.172	0.0000186	N/A	OM1, OM4, OP4	**VUS**
	*KAT6B*	chr10	3C	G123D	0.63	N/A	N/A	OM1, OP1, OP4	**VUS**
	*KAT6B*	chr10	3C	E120K	0.341	N/A	N/A	OM1, OP4,	**VUS**
	*KAT6B*	chr10	3C	C1719Y	0.728	0.0000021	N/A	OP4, OP1	**VUS**
	*KAT6B*	chr10	3A, 13B, 2C, 5C, 6C, 7C	E1096del	N/A	0.1768	0.459676	SBVS1	**B**
	*FANCA*	chr16	1C	Q417 *	N/A	N/A	N/A	OVS1, OM2, OP4	**O**
	*FANCA*	chr16	2C	Q1198 *	N/A	N/A	N/A	OVS1, OM2, OP4	**O**
	*FANCA*	chr16	3C	R24H	N/A	N/A	N/A	OM1, OP4	**VUS**
	*FANCA*	chr16	1C	P1452S	0.372	0.0000123	N/A	OP4	**VUS**
	*FANCA*	chr16	6C	S1088F	0.380	0.0721189	0.0233626	SBS1, SBS2, SBP1	**B**

**Table 4:** The table shows the results of log-rank (Mantel–Cox) test of genes associated with worse survival.

	HR	95% CI of Ratio	*p*-Value	Median Survival with Mutation (Months/Range)	Median Survival Without Mutation (Months/Range)	5-Year Survival (%)
***ATF1***	4.619	0.6676 to 31.96	0.003	8 (1–12)	45 (3–60)	49.5
***FANCA***	3.464	0.7593 to 15.82	0.03	9 (1–30)	42.5 (1–60)	52.6
***MAF***	6.38	0.2294 to 177.4	0.004	4 (3–5)	27.5 (1–60)	45
***NCOA2***	4.855	0.2607 to 90.42	0.02	6 (3–9)	27.5 (1–60)	45
***PIK3CA***	10.6	0.151 to 748	<0.0001	2.5 (2–3)	27.5 (5–60)	45
***TET1***	3.047	0.5937 to 15.64	0.04	8 (1–28)	30 (2–60)	47.4

**Table 5:** Multivariate Cox regression analysis.

Multivariate Analysis			
**Covariates**	**OS**		
**Model 1**	**HR**	**95% CI**	***p*** **-Value**
Peritoneal Carcinomatosis	5.14	1.28–20.60	0.0209
*ATF1*	15.23	2.75–84.37	0.001822
*FANCA*	21.38	3.98–114.79	0.000355
**Model’s likelihood ratio test *p*-value = 3 × 10^−5^**			

## Data Availability

Dataset available on request from the authors.

## Supplementary Materials

The following supporting information can be downloaded at: https://www.mdpi.com/article/10.3390/cancers18091390/s1, Figure S1: Melting curves of microsatellites loci (BAT25, BAT 26, NR21, NR22, NR24, NR27, CAT25, MONO27) in patient 2OC_Group A. The red arrows indicate the unstable markers; Figure S2: Melting curves of microsatellites loci (BAT25, BAT 26, NR21, NR22, NR24, NR27, CAT25, MONO27) in patient 3OC_Group A. The red arrows indicate the unstable markers; Figure S3: Melting curves of microsatellites loci (BAT25, BAT 26, NR21, NR22, NR24, NR27, CAT25, MONO27) in patient 6OC_Group A. The red arrows indicate the unstable markers; Figure S4: Q-RT-PCR; Figure S5: Mutation mapper plots showing the position of amino acid changes corresponding to the variants identified within common mutated genes: *TP53* (A), *ARID1A* (B), *PDE4DIP* (C), *TET2* (D), *RNF213* (E), *ERBB3* (F), *TAF1L* (G); Figure S6: Sanger Sequencing analysis. Sanger electropherograms showing the *TP53* Y220C (A), *TAF1* R869H (B), *TET2* L1721W (C) mutations in cancer samples with matched blood sample; Figure S7: Analysis of OS in mutated genes associated with groups B and C. Kaplan–Meier curves of 5-years survival of HG-SOC patients with mutations in *MAF* (A), *NCOA2* (B), *PIK3CA* (C), *BCL10* (D), *TET1* (E), *FANCA* (F), *ATF1* (G). File S1: Complete demographics and clinical information of the 24 HG-SOC patients enrolled in this study; File S2: NGS metrics sample by sample; File S3: Oncogenicity criteria; File S4: Mutation Frequencies of the 12-Gene Panel in the TCGA Cohort; File S5: List of potential protein-altering somatic variants identified in the HG-SOC samples analyzed.

## Abbreviations

The following abbreviations are used in this manuscript:

ATF1	Activating Transcription Factor 1
BRCA	Breast Cancer Gene
bZIP	Leucine Zipper Domain of the Basic Region
DOT1L	DOT1-Like Histone Lysine Methyltransferase
FANCA	Fanconi Anemia Complementation Group A
FANCC	Fanconi Anemia Complementation Group B
FIGO	International Federation of Gynecology and Obstetrics
H15	Histone H1/H5-Like Domain
HG-SOC	High-Grade Serous Ovarian Carcinoma
KAT6B	Lysine Acetyltransferase 6B
LB	Likely Benign
LO	Likely Oncogenic
MAF	V-Maf Musculoaponeurotic Fibrosarcoma Oncogene Homolog
MLLT10	Mixed Lineage Leukemia Translocated To 10 (AF10)
NCOA2	Nuclear Receptor Coactivator 2
NLS	Nuclear Localization Signal
NRAS	Neuroblastoma RAS Viral Oncogene Homolog
PIK3CA	Phosphatidylinositol-4,5-Bisphosphate 3-Kinase Catalytic Subunit Alpha
REVEL	Rare Exome Variant Ensemble Learner
SNVs	Single Nucleotide Variants
SRC	Steroid Receptor Coactivator Domain
TAF1	TATA-Box Binding Protein Associated Factor 1
TCGA	The Cancer Genome Atlas
TET1	Ten-Eleven Translocation 1
TOP1	DNA Topoisomerase I
VUS	Variant of Uncertain Significance
