# Supplementary material for: Genomic Predictors of Platinum Resistance and Survival in High-Grade Serous Ovarian Carcinoma: Insights from an Explorative Targeted Next-Generation Sequencing Analysis
Source: Cancers (Basel). 2026 Apr 28;18(9):1390. doi: 10.3390/cancers18091390 (PMC13163086; doi:10.3390/cancers18091390)
Supplement: Supplementary file 1 [file cancers-18-01390-s001.zip › cancers-4207418-supplementary.pdf]

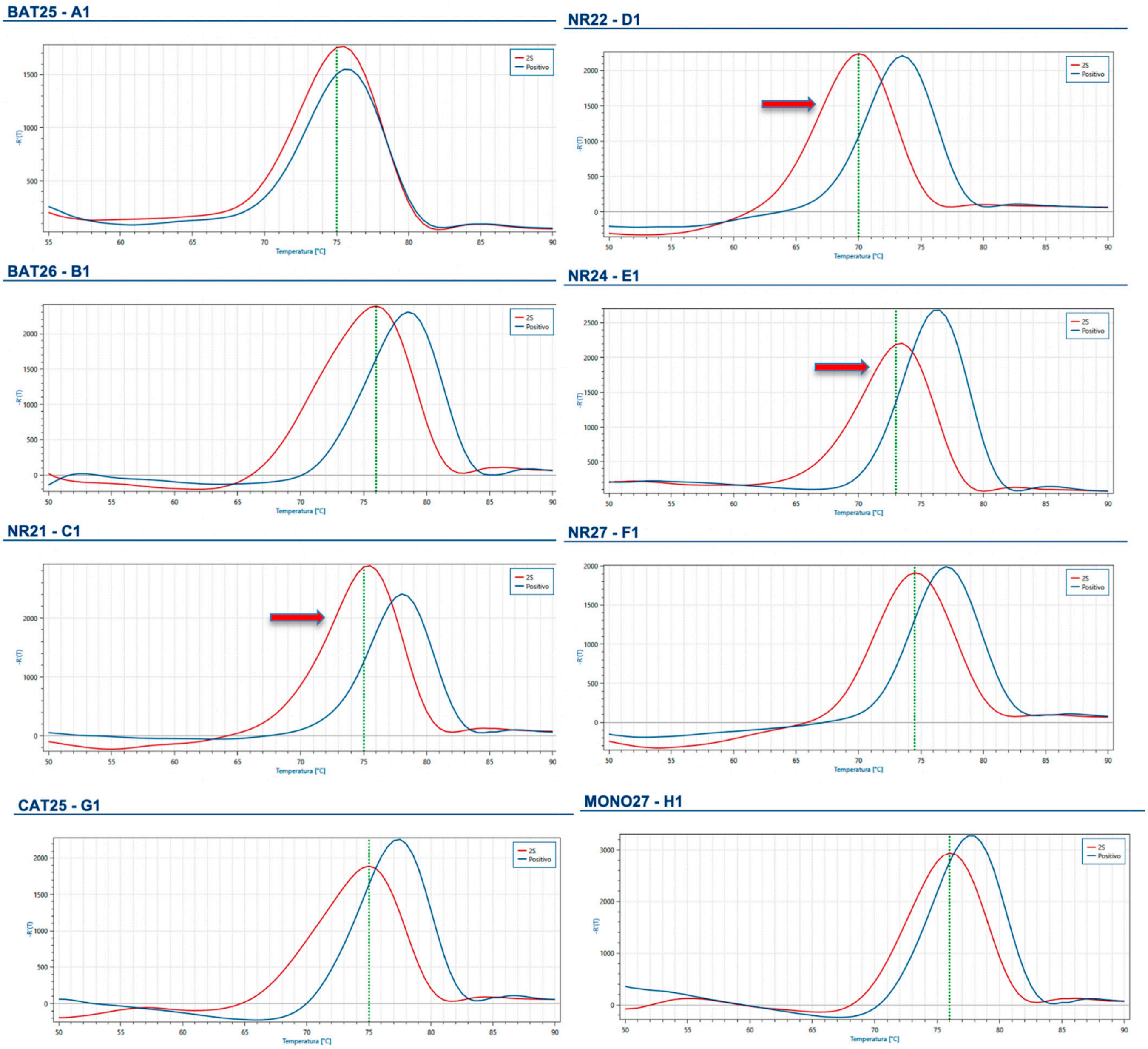

Figure S1. Melting curves of microatellites loci (BAT25, BAT 26, NR21, NR22, NR24, NR27, CAT25, MONO27) in patient 2OC_Group A. The red arrows indicate the unstable markers.

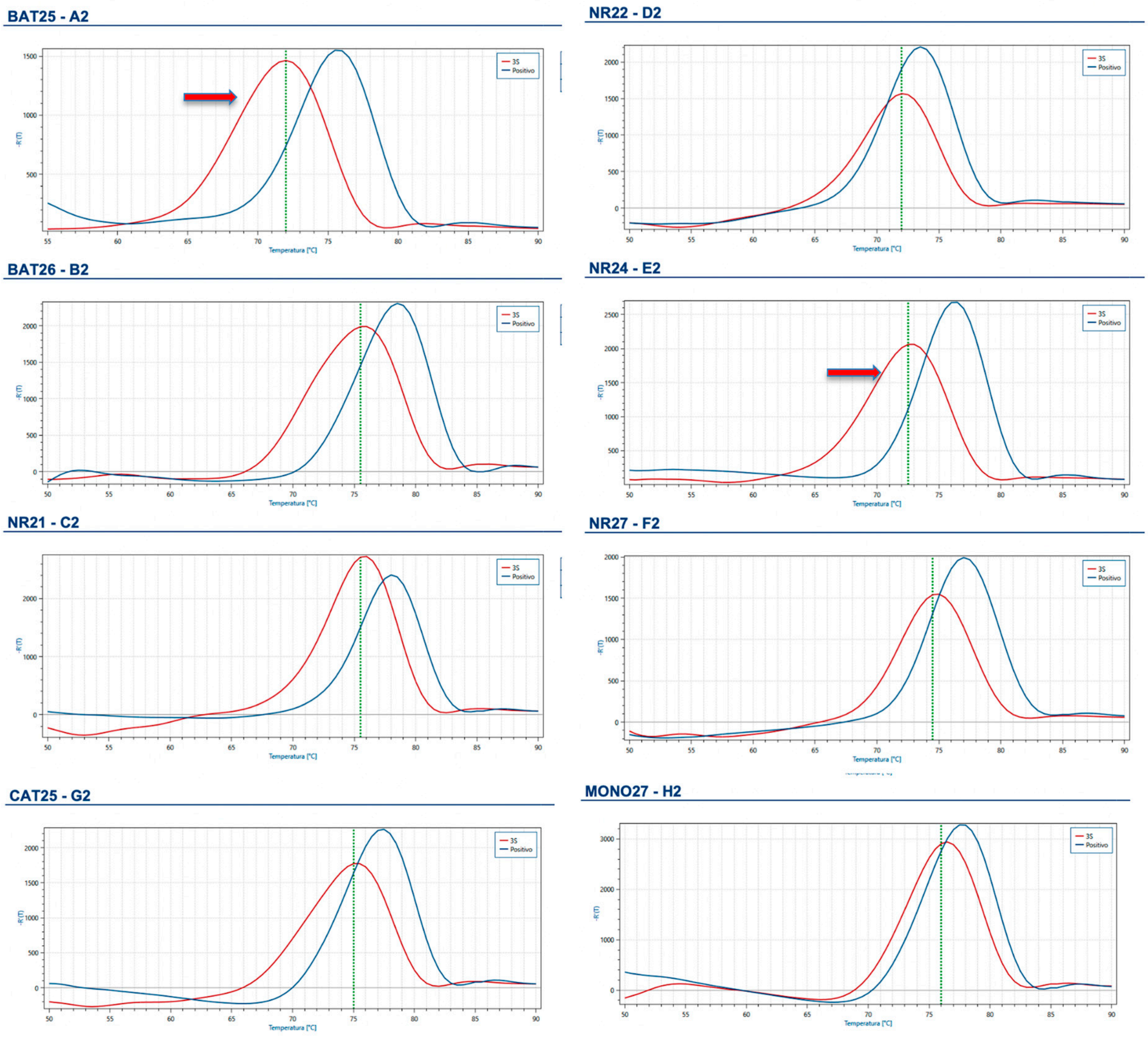

Figure S2. Melting curves of microatellites loci (BAT25, BAT 26, NR21, NR22, NR24, NR27, CAT25, MONO27) in patient 3OC_Group A. The red arrows indicate the unstable markers.

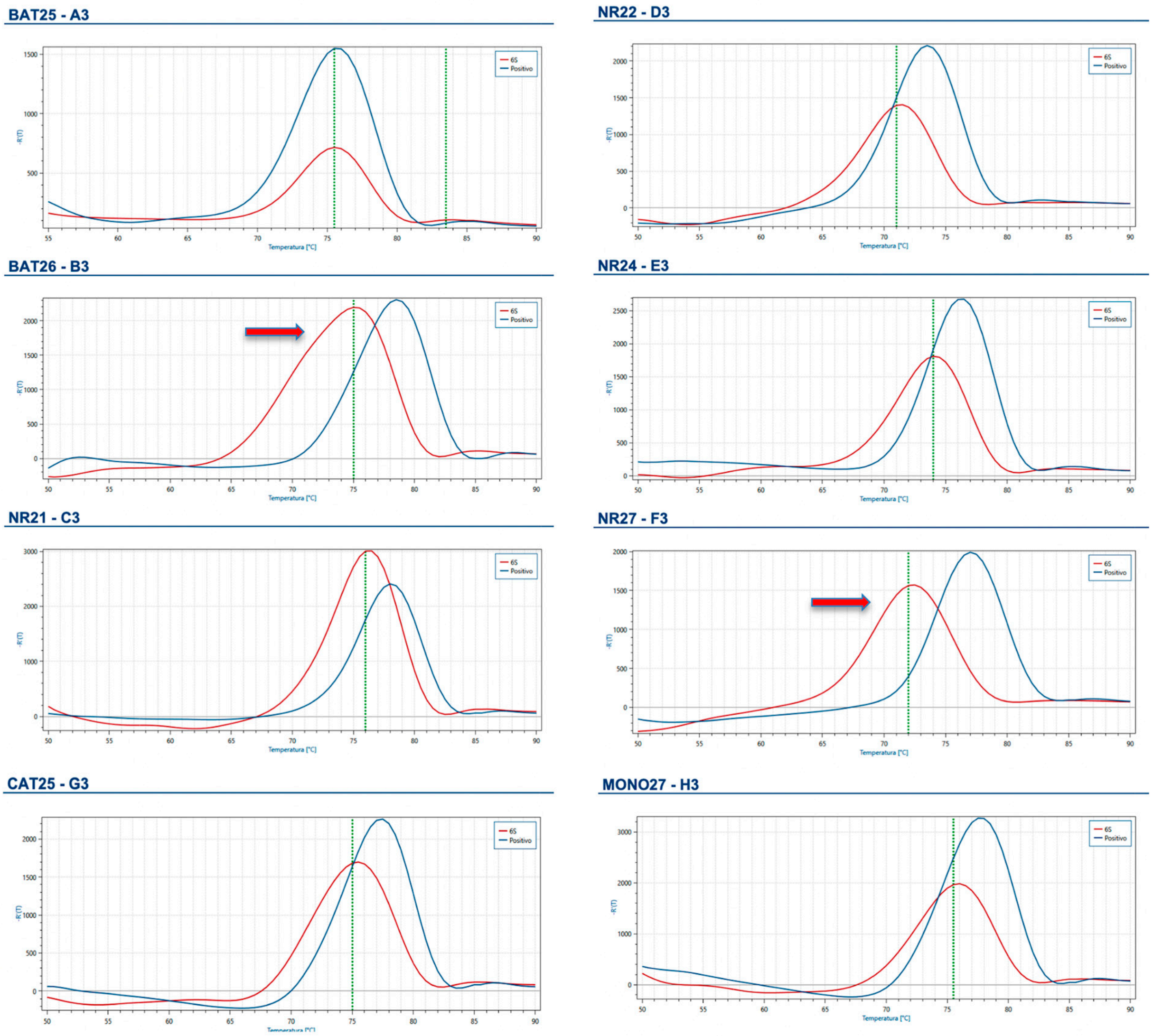

Figure S3. Melting curves of microatellites loci (BAT25, BAT 26, NR21, NR22, NR24, NR27, CAT25, MONO27) in patient 6OC_Group A. The red arrows indicate the unstable markers.

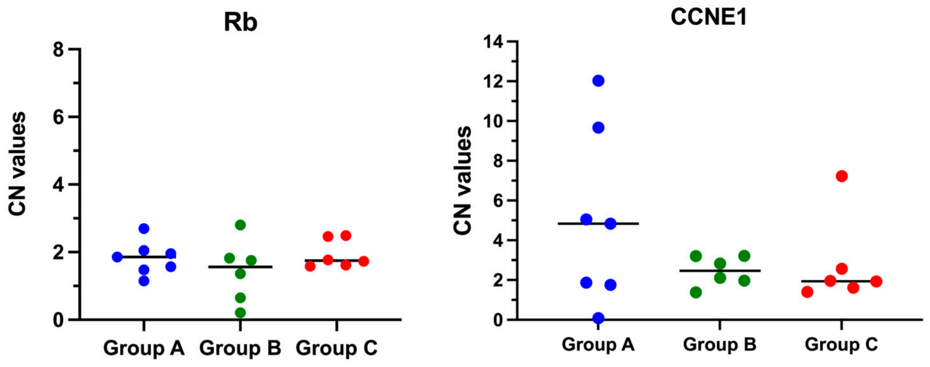

Figure S4. Q-RT-PCR analysis of RB1 and CCNE1 gene copy number. Gene copy number values were normalized to a diploid reference, with the copy number set to 2.

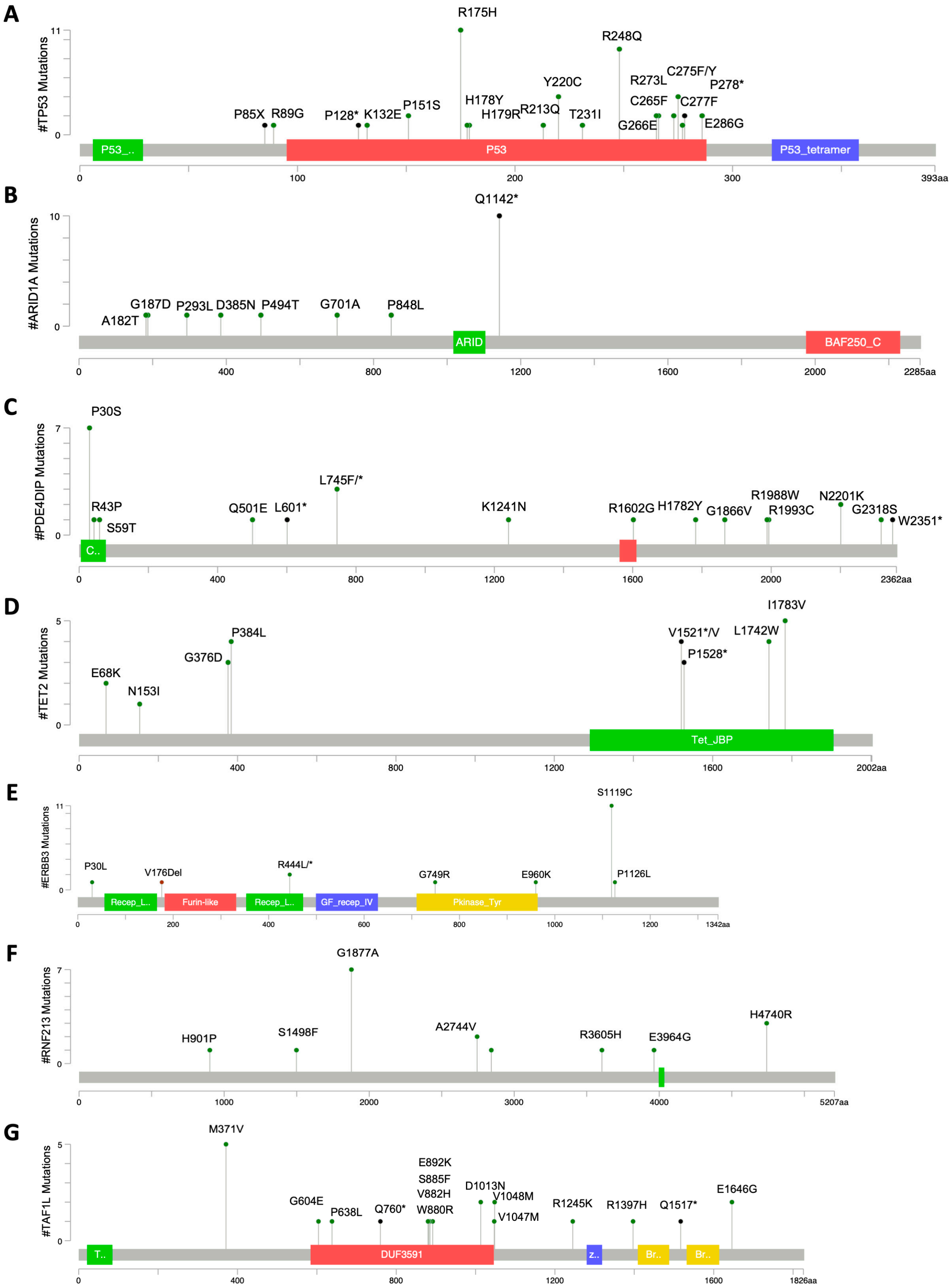

Figure S5. Mutation mapper plots showing the position of amino acid changes corresponding to the variants identified within common mutated genes: *TP53* (**A**), *ARID1A* (**B**), *PDE4DIP* (**C**), *TET2* (**D**), *RNF213* (**E**), *ERBB3* (**F**), *TAF1L* (**G**). Mutation mapper plots were created with: https://www.cbioportal.org/mutation_mapper on 08 April 2026.

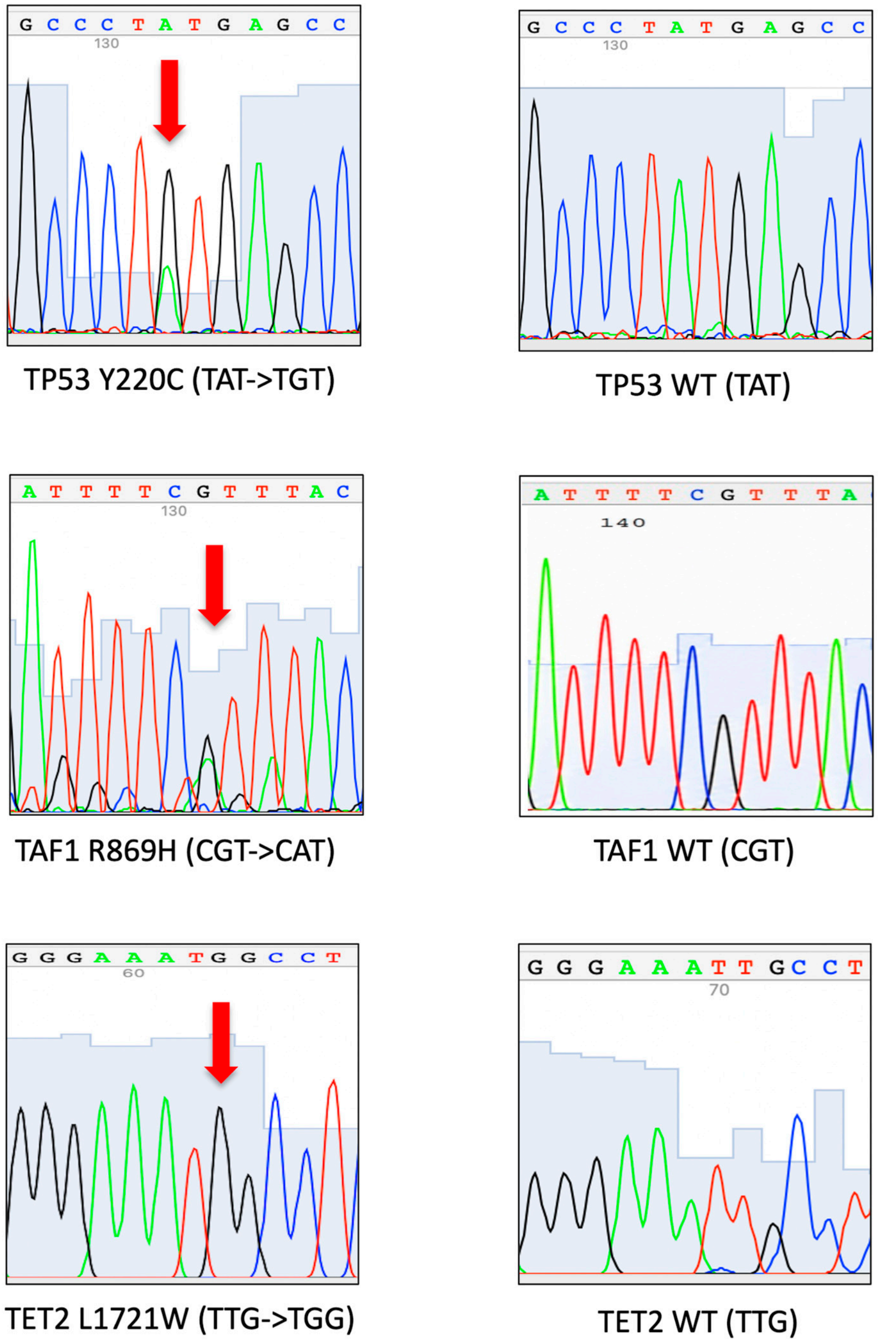

Figure S6. Sanger Sequencing analysis. Sanger electropherograms showing the *TP53* Y220C (A), *TAF1* R869H (B), *TET2* L1721W (C) mutations in cancer samples with matched blood sample.

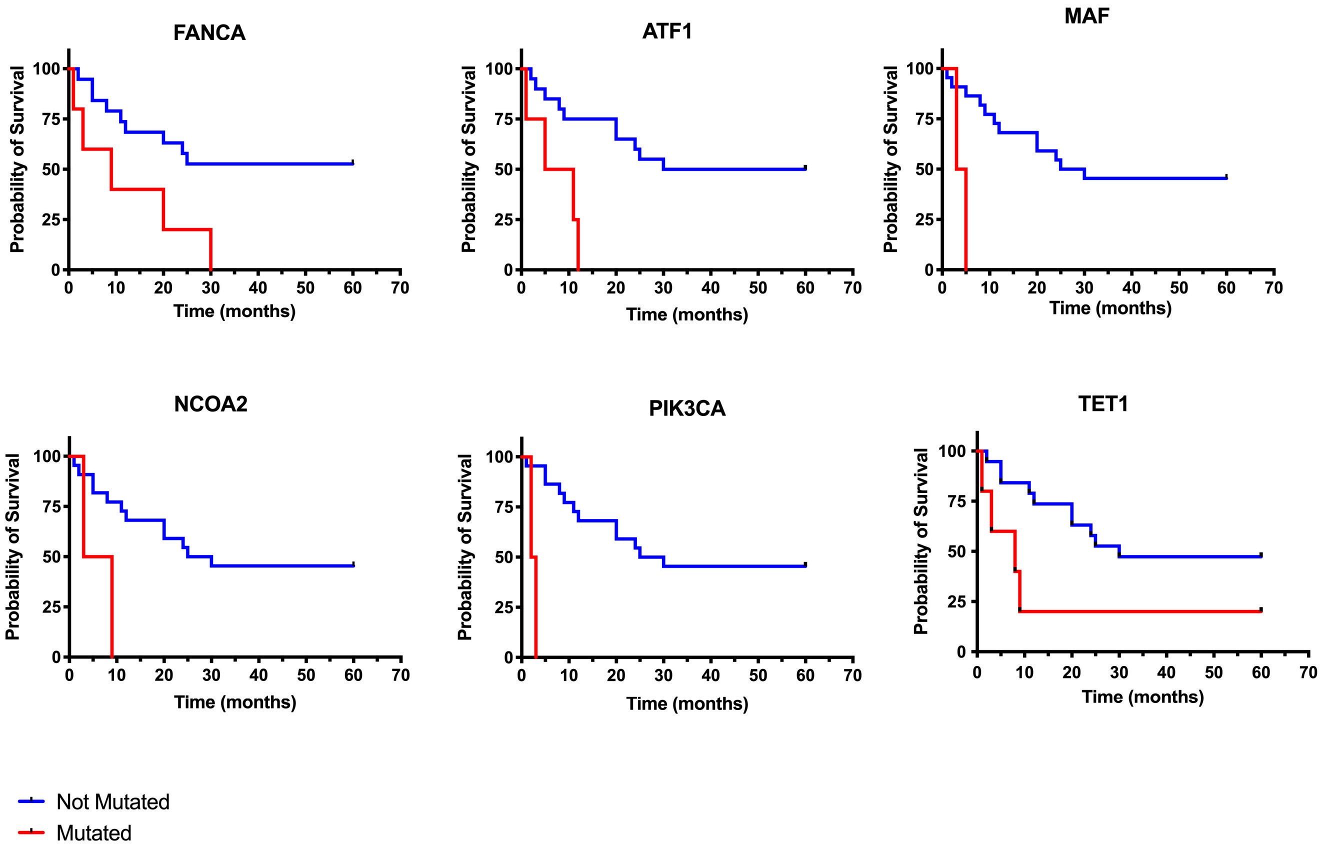

Figure S7. Analysis of OS in mutated genes associated with Groups B and C. Kaplan-Meier curves of 5-years survival of HG-SOC patients with mutations in *FANCA* (A), *ATF1* (B), *MAF* (C), *NCOA2* (D), *PIK3CA* (E), *TET1* (F).
